# Zero-shot segmentation using embeddings from a protein language model identifies functional regions in the human proteome

**DOI:** 10.1371/journal.pcbi.1012929

**Published:** 2025-11-11

**Authors:** Ami G. Sangster, Cameron Dufault, Haoning Qu, Denise Le, Julie D. Forman-Kay, Alan M. Moses

**Affiliations:** 1 Cell & Systems Biology, University of Toronto, Toronto, Ontario, Canada; 2 Department of Computer Science, University of Toronto, Toronto, Ontario, Canada; 3 Molecular Medicine Program, Hospital for Sick Children, Toronto, Ontario, Canada; 4 Department of Biochemistry, University of Toronto, Toronto, Ontario, Canada; Tel Aviv University, ISRAEL

## Abstract

The biological function of a protein is often determined by its distinct functional units, such as folded domains and intrinsically disordered regions. Identifying and categorizing these protein segments from sequence has been a major focus in computational biology which has enabled the automatic annotation of folded protein domains. Here we show that embeddings from the unsupervised protein language model ProtT5 can be used to identify and categorize protein segments without relying on conserved patterns in primary amino acid sequence. We present Zero-shot Protein Segmentation (ZPS), where we use embeddings from ProtT5 to predict the boundaries of protein segments without training or fine-tuning any parameters. We find that ZPS boundary predictions for the human proteome are better at reproducing reviewed annotations from UniProt than established bioinformatics tools and ProtT5 embeddings of ZPS segments can categorize over 200 of the most common UniProt annotations in the human proteome, including folded domains, sub-domains, and intrinsically disordered regions. To explore ZPS predictions, we introduce a new way to visualize protein embeddings that closely resembles diagrams of distinct functional units in protein biology. Since ZPS and segment embeddings can be used without training or fine-tuning, the approach is not biased towards known annotations and can be used to identify and categorize unannotated protein segments. We used the segment embeddings to identify unannotated mitochondrion targeting signals and SYGQ-rich prion-like domains, which are functional regions within intrinsically disordered regions. We expect that the analysis of protein segment embedding similarity can lead to valuable information about protein function, including about intrinsically disordered regions and poorly understood protein regions.

## Author summary

Understanding protein function has been a major focus of computational biology for decades. Classical approaches have used amino acid sequences and compositional biases that are conserved over evolution to identify protein segments that are associated with specific biological functions. Our results put forward a new approach for identifying protein segments which can be associated with specific biological functions. This approach applies zero-shot segmentation to protein language model embeddings and does not require any training or fine-tuning, so it has the potential to generalize to rare and poorly annotated protein segments. We also present a new approach for visualizing protein language model embeddings using colours to indicate the similarity of protein segments in the embedding space.

## Introduction

Proteins can contain a variety of distinct functional units including independently folding regions, known as folded domains, and regions lacking stable structure, such as intrinsically disordered regions (IDRs). Together, these distinct functional units determine the overall function of the protein. Powerful bioinformatics tools, such as Pfam [1] and Prosite [2], are widely used to identify and categorize folded domains from amino acid sequences alone. Other approaches are applied to protein structures to identify folded domains within proteins, referred to as protein segmentation [3,4]. In addition to folded domains, IDRs are present in over 60% of human proteins [5], perform important functions [6], and can be identified directly from amino acid sequences with high accuracy [7]. Unlike folded domains, functional categorization of IDR types remains challenging, though recent works have shown some success using conserved sequence properties [8,9] and supervised deep learning [10,11]. Unsupervised bioinformatics approaches, such as fLPS2 [12] and a Chi-Score Analysis [13], can segment proteins more generally based on statistical properties of amino acid sequences. These approaches can distinguish IDRs from domains and have revealed a non-random modular architecture within IDRs [13].

Here we explore the use of Protein Language Models (pLMs) to identify and categorize folded domains and IDRs from the human proteome. We use the pLM ProtT5 [14] to encode protein sequences into high-dimensional vectors known as embeddings. ProtT5 was pre-trained through unsupervised learning, a process where the model predicts masked (or hidden) amino acids in protein sequences and therefore does not require labelled data. Performing this task requires ProtT5 to develop contextualized embeddings of each amino acid based on the surrounding protein sequence, which are then used to predict the masked amino acids. After pre-training, visualizations of dimensionally reduced embeddings can reveal associations with amino acid properties, structure, and evolutionary relationships [14–16]. Additionally, pLMs can be used as a starting point for further training, known as fine-tuning, or the embeddings from a pre-trained model can be used as input to train another model. Both options have been applied to a variety of protein related tasks [15–20], including amino acid level domain annotation [21].

Embeddings or pLMs can also be used to make predictions on a new task, different from the pre-training task, without training or fine-tuning on the new task. This is referred to as “zero-shot” prediction. Protein embeddings have been used for zero-shot prediction of variant effects [22,23] and drug-target binding [24]. Zero-shot applications offer the potential for biological discovery without being tied to the original training task or being biased by the content and availability of training data. Recent work in image analysis has demonstrated that an unsupervised vision transformer can identify the boundaries of objects in images without human labelled examples; this is referred to as zero-shot image segmentation [25,26]. Inspired by this work, we sought to test whether a transformer-based pLM, ProtT5 [14], can perform zero-shot protein segmentation.

Here, we demonstrate that a change point analysis applied to ProtT5 embeddings can identify the boundaries of biologically meaningful protein segments. We achieved this without training or fine-tuning any parameters, so we refer to this as Zero-shot Protein Segmentation (ZPS). We compare ZPS boundary predictions to annotations from UniProt [27], a protein annotation database which curates annotations from the literature and combines annotations from other databases and bioinformatics tools such as MobiDB [28] and ProRule [2]. We show that for the human proteome, the segments defined by ZPS can reproduce the boundaries of annotations from MobiDB for disorder and compositional biases more closely than other unsupervised methods designed for these types of protein sequences [12,13]. Furthermore, we find that the ProtT5 embeddings of protein segments defined by ZPS can be used to categorize UniProt annotations (n>=25), including domains, sub-domains, and different types of IDRs. Finally, as a specific application of these approaches, we identify unannotated mitochondrion targeting signals and SYGQ-rich prion-like domains in the human proteome.

## Results

### Known domains, IDRs, and motifs are found within ProtT5 embedding of FUS

**Fig 1:**
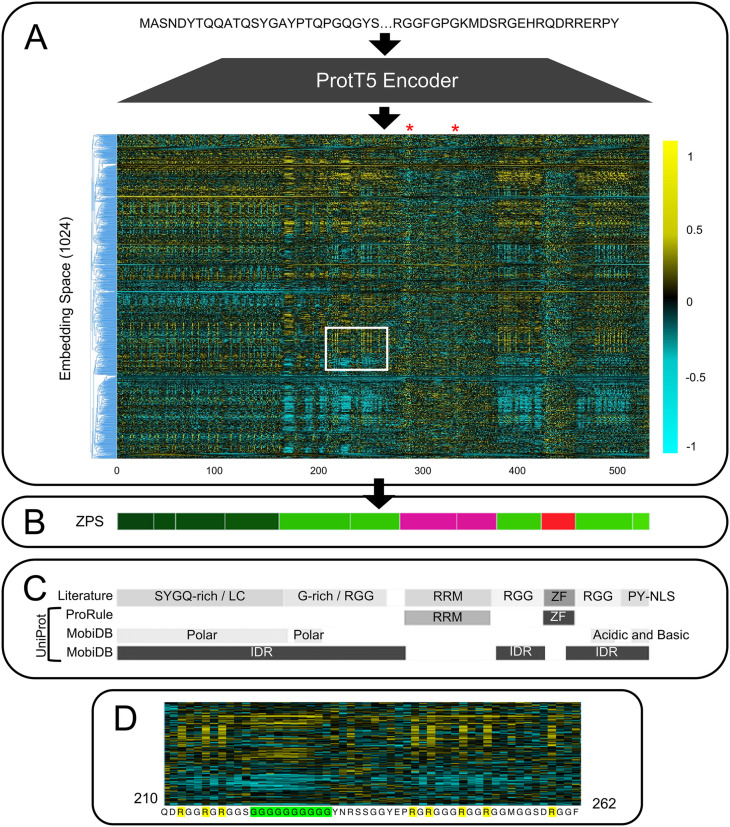
Zero-Shot Protein Segmentation Summary and Comparison to the Literature. (A) A per-residue protein embedding from ProtT5 for human protein FUS (UniProt ID: P35637). The embedding is visualized as a heatmap where the x-axis represents each amino acid along the proteins sequence (which is maintained across the panels in this figure), and the y-axis is the ProtT5 embedding space. The embedding space is ordered by hierarchical agglomerate clustering to make patterns in the embedding space more visible. RNP1 and RNP2 motifs are indicated with a red asterisk above the protein embedding. The white box indicates the portion of the heatmap shown in panel D. (B) Segments of FUS found using Zero-shot Protein Segmentation (ZPS). The colour of each protein segment was obtained by reducing 1024-dimensional segment embeddings to 3-dimensions, which were scaled to RGB colours (see Methods). (C) FUS annotations from the literature (see Methods) and UniProt. MobiDB and ProRule annotations were retrieved from UniProt. (D) A small portion of protein embedding heatmap showing the first RGG region in FUS (amino acids 210-262). This highlights patterns in this section of the protein embedding that emphasize arginine (R, highlighted in yellow) and glycine (G, highlighted in green).

To investigate whether ProtT5 embeddings can be used to identify the boundaries of biologically meaningful protein segments, we first visualized the ProtT5 embedding of human RNA-binding protein FUS [14] as a heatmap (Fig 1A). We selected FUS because it is a well-studied protein with a combination of annotated folded domains and IDRs. In the heatmap, we observed distinct segments in the embedding along the protein’s amino acid sequence. We identified boundaries of these segments using a change point analysis and used these boundaries to define protein segments, an approach we refer to as Zero-shot Protein Segmentation (ZPS), then visualized them (Fig 1B). The segments defined by ZPS closely matched with annotations from UniProt and the literature [29,30] (Fig 1C).

We noted that in addition to known boundaries, the embedding also appears to contain higher resolution information than its annotations (Fig 1). For example, the RNA Recognition Motif (RRM) domain (folded) is broken into two segments by ZPS. Generally, RRM domains have the structure **β**_**1**_α_1_β_2_**β**_**3**_α_2_β_4_ with an RNP2 motif in **β**_**1**_ and an RNP1 motif in **β**_**3**_ [31] which align with the 2 faint vertical bands within the embedding of the RRM domain (Fig 1A, red asterisks, amino acids 287–293 and 333–341). Each of the two ZPS segments within the RRM domain begins with one of these motifs, suggesting that the pLM is distinguishing a real functional and structural boundary within the RRM domain. Similarly, within the IDRs, arginine-glycine-glycine (RGG) regions are defined by multiple short repeating motifs of arginine and glycine and are associated with specific biological functions [32]. In the RGG regions of the FUS embedding, the bold vertical lines are the arginines and the thicker vertical bands are the glycines (Fig 1A and 1D, amino acids 161–279, 376–419, 453–509). These results are consistent with previous observations that pLMs learn key functional residues and regions within proteins [15,22,33] and indicates that this observation applies to both folded domains and IDRs.

For a more compact and interpretable visualization of the high-dimensional segment embeddings, we display each segment as a block of colour along the linear protein sequence (Fig 1B), as commonly seen in the molecular biology literature [29,30,32,34]. We achieve this by averaging the per-residue embeddings over the sequence length of a ZPS protein segment to obtain a “segment embedding” (see Methods). Then the segment embedding is reduced from a 1024-dimensional space to a 3-dimensional RGB colour space to obtain a specific colour for that segment (S1 Fig). Just as distinct clusters in a 2-dimensional visualization of protein embeddings can be associated with biologically meaningful annotations (such as domains, evolutionary relationships, and folded structures) [15,16,19,35], distinct colours in the 3-dimensional RGB space can be associated with biologically meaningful annotations from the literature (Fig 1). For example, there are SYGQ-rich and RGG regions within the N-terminal IDR of FUS that are easily distinguishable by their segment colour and well-defined in the literature, but these are not well-characterized in UniProt (Fig 1). Despite over-segmentation relative to annotations from UniProt and the literature, segments with the same colour fall under the same annotation, suggesting that segment embeddings in the high-dimensional space can be associated with annotations (explored further in later sections).

### Segment embeddings can be used to categorize annotated regions of RNA binding proteins

**Fig 2:**
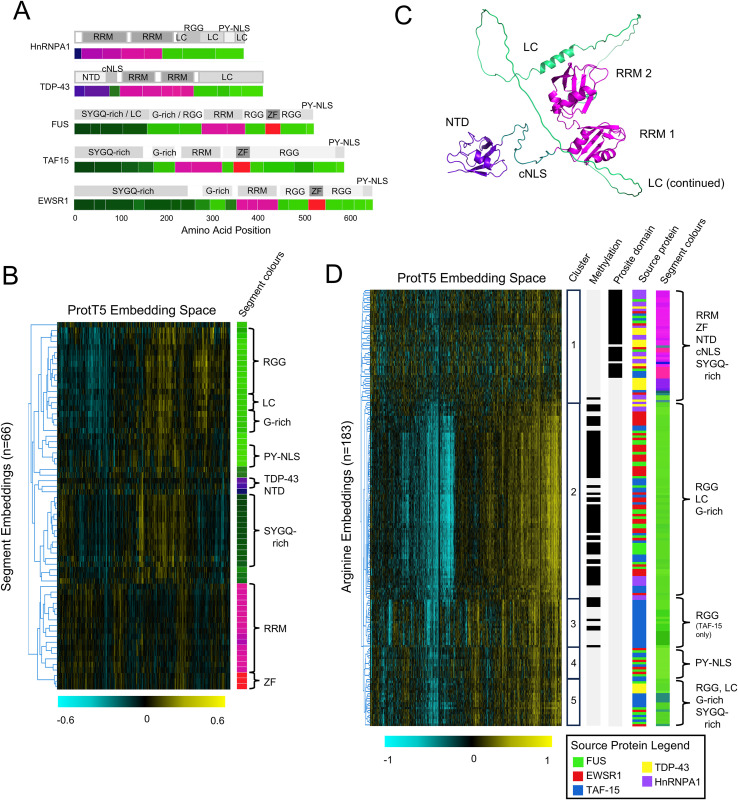
Segment Embeddings and Segment Colours of RNA-Binding Proteins. (A) Segment embedding boundaries and colours compared to annotations from the literature (see Methods) for the FET family (FUS, EWSR1 (UniProt ID: Q01844), and TAF-15 (UniProt ID: Q92804)), HnRNPA1 (UniProt ID: P09651), and TDP-43 (UniProt ID: Q13148). (B) Segment embeddings shown in panel A were ordered by hierarchical agglomerate clustering and visualized as a heatmap. Corresponding colours of the segment embeddings are shown to the right of the heatmap and labelled with annotations from the literature. (C) AlphaFoldDB structure of TDP-43 showing segment colours. Abbreviations for folded domains labelled here include RNA Recognition Motifs (RRM), N-terminal domain (NTD), and Zinc Fingers (ZF). Abbreviations for IDR sub-regions labelled here include canonical Nuclear Localization Signal (cNLS), Low Complexity (LC) regions, PY-motif Nuclear Localization Signals (PY-NLS), and RGG (arginine-glycine-glycine motif) regions. G-rich and SYGQ-rich regions describe compositional biases that define sub-regions of IDRs. (D) Arginine embeddings from the FET family, TDP-43, and HnRNPA1 proteins were ordered by hierarchical agglomerative clustering and shown in a heatmap. To the right of the heat map (and moving towards the right), we show the numbered clusters, which arginines are annotated as methylation sites on UniProt (black for methylated), which arginines are contained within a ProRule domain annotation on UniProt (black for domain), which protein the arginine originated from (FUS in green, EWSR1 in red, TAF-15 in blue, TDP-43 in yellow, and HnRNPA1 in purple), the colour of the ZPS segment that the arginine originated from, and the names of the domains and regions that the arginines in each cluster originated from.

To assess whether segment embeddings can be used to categorize segments across multiple proteins, we segmented well-characterized RNA binding proteins using ZPS then clustered their segment embeddings and projected their segment colours onto AlphaFoldDB structures [36]. We selected the FET family (FUS, EWSR1, and TAF15), TDP-43, and HnRNPA1 which have a variety of annotated folded domains, IDRs, and regions within IDRs. The ZPS boundaries are consistent with annotations from the literature [29,30] and segments with the same annotation have similar segment embeddings, as visualized by their similar segment colours (Fig 2A). Further, clustering the high-dimensional segment embeddings of these proteins separates the visually similar nuclear localization signals from RGGs (Fig 2B), consistent with the different annotations of these regions. Clustering the segment embeddings (Fig 2B) also shows that the two N-terminal segments of TDP-43 (Fig 2, purple) that comprise the N-terminal domain [34] are more similar to IDRs (Fig 2, green) than folded domains (Fig 2 pink and red), despite AlphaFoldDB displaying a confident structure (pLDDT>90) (Fig 2C, purple). Surprisingly, this is consistent with the literature since the stability of this structure is debated [37,38].

To further investigate amino acids in the embedding space that are visually distinct, such as the amino acids in the RGG regions (Fig 1D), we clustered all the arginines in the FET family, TDP-43, and HnRNPA1 (Fig 2D). Cluster 2 contains 83 arginines from a variety of different annotationed regions including RGG-s, low complexity (LC), and G-rich, 61 (73%) of which are annotated as methylation sites on UniProt. We tested the percent of methylated arginines in this cluster by shuffling annotated methylation sites within proteins and within annotated regions of proteins. We found that 73% is greater than the 49% expected by chance when shuffling methylation sites within arginines of the same protein (n = 10,000, Z-score = 7.55, empirical P-value = < 1/10,000) as well as the 66% expected by chance of shuffling within annotated regions (n = 10,000, Z-score = 3.48, empirical P-value 10/10,000). Additionally, cluster 5 contains arginines from the same annotated regions as cluster 2, but none of these arginines are known to be methylated. Interestingly, cluster 3 only contains arginines from RGG regions in TAF-15, cluster 4 only contains arginines from PY-NLS of the FET family, and cluster 1 contains all arginines from structured domains as well as a few from the NTD, cNLS, and SYGQ-rich regions. This demonstrates that the contextualized embeddings of arginine amino acids contain important functional information about IDRs, such as whether they are methylation sites.

Taken together these results suggest that the similarity of segment embeddings could be used to simultaneously categorize different folded domains and different IDR sub-regions across multiple proteins (explored further in later sections). Additionally, the above analysis demonstrates that visualizing protein embeddings as segments in the 3-dimensional colour space offers a simple strategy for insight into unsupervised pLM embeddings.

### Zero-shot protein segmentation can reproduce the boundaries of UniProt annotations from the human proteome

To quantitatively assess the boundaries predicted by ZPS, we systematically compared segment boundaries to annotated segments in the human proteome from UniProt. We compare ZPS to Pfam [1] and PrositeScan [2], which are supervised bioinformatics tools that primarily identify folded domains in proteins. We also compare ZPS to fLPS2 [12] and Chi-Score Analysis [13], which are unsupervised bioinformatics tools that segment proteins. Additionally, we distinguish between UniProt, MobiDB, and ProRule, where MobiDB and ProRule are separate databases that are included in UniProt. MobiDB contains IDR and compositional biases annotations while ProRule contains domain annotations.

First, we evaluated this approach as a segmentation problem using Intersection over Union (IoU), a widely used measure of segmentation agreement from image analysis [25,39]. A measure similar to IoU was previously proposed for assessing protein secondary structure prediction of proteins [40]. We find that when evaluated on all human proteome annotations in UniProt (which includes both MobiDB and ProRule), ZPS outperforms both the supervised and unsupervised bioinformatics approaches on average IoU (pos) and recall (Table 1). This is consistent with our results for FUS and other proteins (Figs 1 and 2), where ZPS can identify the boundaries of both folded domains and IDRs. We find that ZPS outperforms both supervised and unsupervised bioinformatics approaches on average IoU (pos) and recall for annotations from MobiDB (Table 1). Finally, for ProRule annotations we find that ZPS performance falls between supervised and unsupervised bioinformatics approaches, where as expected, supervised bioinformatics approaches trained on domains have the best performance (Table 1).

**Table 1:** Segmentation Evaluation for Human Proteome Segment Annotations from UniProt.

	All UniProt (N = 92k)					MobiDB (N = 23k)					Prosite (N = 22k)				
	Pred. seg.	AIoU (pred)	AIoU (pos)	Prec.	Rec.	Pred. seg.	AIoU (pred)	AIoU (pos)	Prec.	Rec.	Pred. seg.	AIoU (pred)	AIoU (pos)	Prec.	Rec.
***Supervised***															
Pfam	59k	0.466	0.364	0.497	0.320	29k	0.033	0.047	0.018	0.023	40k	0.388	0.711	0.435	0.793
Prosite Scan	42k	**0.638**	0.363	**0.635**	0.289	24k	0.111	0.137	0.097	0.098	33k	**0.570**	**0.879**	**0.576**	**0.853**
***Unsupervised***															
fLPS2	109k	0.268	0.342	0.204	0.242	68k	0.152	0.382	0.112	0.326	61k	0.123	0.299	0.075	0.205
fLPS2 (low complexity)	48k	0.247	0.148	0.198	0.102	37k	**0.206**	0.328	**0.171**	0.271	28k	0.055	0.079	0.043	0.053
Chi-Score Analysis	554k	0.107	0.340	0.036	0.215	317k	0.051	0.429	0.026	0.352	310k	0.054	0.278	0.009	0.120
Chi-Score (filtered)	81k	0.336	0.375	0.299	0.265	51k	0.177	0.391	0.146	0.316	43k	0.161	0.332	0.129	0.253
***Our Method***															
ZPS	253k	0.231	**0.525**	0.172	**0.472**	152k	0.107	**0.580**	0.088	**0.569**	144k	0.123	0.534	0.077	0.497
ZPS (corrected)	164k	0.282	0.509	0.230	0.411	98k	0.129	0.527	0.111	0.466	93k	0.158	0.541	0.120	0.505

We report the number of predicted segments (Pred. seg.) and Intersection over Union (IoU) metrics such as Average IoU (AIoU) with respect to the predicted (pred) and positive (pos) annotations (see Methods) Precision (Prec.), and Recall (Rec.). ZPS (corrected) uses a simple correction for over-segmentation (see Methods). The best performance for each measure is shown in **bold.** N shows the number of annotated segments in the dataset. We use an IoU threshold of 0.5 to define precision and recall.

Since we have previously observed over-segmentation relative to annotations from UniProt and the literature (Figs 1 and 2), we also report metrics for over-segmentation corrected ZPS (see Methods), which improves precision and reduces recall (Table 1). However, ZPS and ZPS corrected do not have the conventional precision-recall trade-off, so we cannot simply look at ZPS at a different score threshold to get a higher precision at the cost of recall. For ZPS and ZPS corrected, the precision stays constant as recall increases (S1 Fig). This is in part due to the complications of using conventional confusion matrix-based metrics on a segmentation problem and that UniProt represents a very large but still incomplete set of protein annotations.

In addition to IoU, we assessed the predicted boundaries by calculating the distance in amino acids between the boundaries of annotated segment and predicted segment pairs (see Methods). Overall, we found that ~40–50% of ZPS boundaries are less than 10 amino acids from a known boundary (Table 2). This demonstrates that on average, ZPS can accurately predict about half the boundaries of known segment annotations. Furthermore, ZPS outperforms supervised and unsupervised bioinformatics tools by about 10–30% when we consider all human proteome annotations in UniProt or only this subset from MobiDB. As expected, Prosite Scan and Pfam perform best on the ProRule annotations from UniProt. Nevertheless, a change point analysis on ProtT5 embeddings can consistently outperform unsupervised bioinformatics tools developed with specific biological knowledge for IDRs and compositional biases. Furthermore, unlike other methods, our approach shows reasonable performance on both folded domains and IDRs in the human proteome.

**Table 2:** Boundary Evaluation for Human Proteome Segment Annotations from UniProt.

	All Uniprot (N = 92k)			MobiDB (N = 23k)			ProRule (N = 22k)		
	Predicted Segments	Missed Uniprot Segments	<10 amino acids	Predicted Segments	Missed MobiDB Segments	<10 amino acids	Predicted Segments	Missed ProRule Segments	<10 amino acids
***Supervised***									
Pfam	59k	31k	26.31%	29k	18k	2.40%	40k	1k	58.02%
Prosite Scan	42k	38k	31.23%	24k	15k	9.44%	33k	0.5k	**82.75%**
***Unsupervised***									
fLPS2	109k	7k	19.15%	68k	0.9k	27.72%	61k	2k	14.06%
fLPS2 (low complexity)	48k	52k	12.51%	37k	6k	28.42%	28k	16k	6.92%
Chi-Score Analysis	554k	11k	28.06%	317k	3k	37.14%	310k	4k	19.98%
Chi-Score (filtered)	81k	11k	23.40%	51k	3k	30.07%	43k	4k	15.84%
***Our Method***									
ZPS	253k	1k	**41.36%**	152k	0.3k	**48.51%**	144k	0.3k	38.62%
ZPS (corrected)	164k	1k	37.08%	98k	0.3k	41.32%	93k	0.3k	35.61%

We report the number of predicted segments, the number of annotated segments without an overlapping predicted segment (Unpaired Segments), and the percentage of annotated boundaries with a predicted boundary less than 10 amino acids away. ZPS (corrected) uses a simple correction for over-segmentation (see Methods). The best performance for each measure is shown in **bold.** N shows the number of annotated segments in the dataset.

### Segment embeddings can distinguish UniProt annotations

Our results for well-characterized RNA binding proteins (Fig 2) suggested that segment embeddings can distinguish different types of regions within proteins. So, we tested whether segment embedding similarity can simultaneously categorize the different types of annotations available for the human proteome on UniProt (n>= 25). We used the labels of the 1-nearest neighbour (1-nn) in the high dimensional segment embedding space as predictions for each segment, as is standard practice in evaluating unsupervised deep learning methods [26,41]. For this evaluation we allowed each segment to have multiple labels (multi-label classification), adopting any annotation that covers at least 30% of the segment (see Methods), then performed a binary evaluation for each label. This included over 150,000 protein segments with 224 different labels. To confirm that segment embeddings from the T5 model are more informative than embeddings of simple amino acid sequences, we compare our results to 3-mer embeddings of the same protein segments (see Methods). The average precision for ZPS is 0.772 (+/- 0.003) compared to an average precision of 0.573 (+/-0.003) for 3-mers embeddings (S1 Table). This demonstrates the segment embedding similarity can distinguish different types of protein segments including structured domains, IDRs, and others such as coiled-coils, from unsupervised pre-training alone.

**Fig 3:**
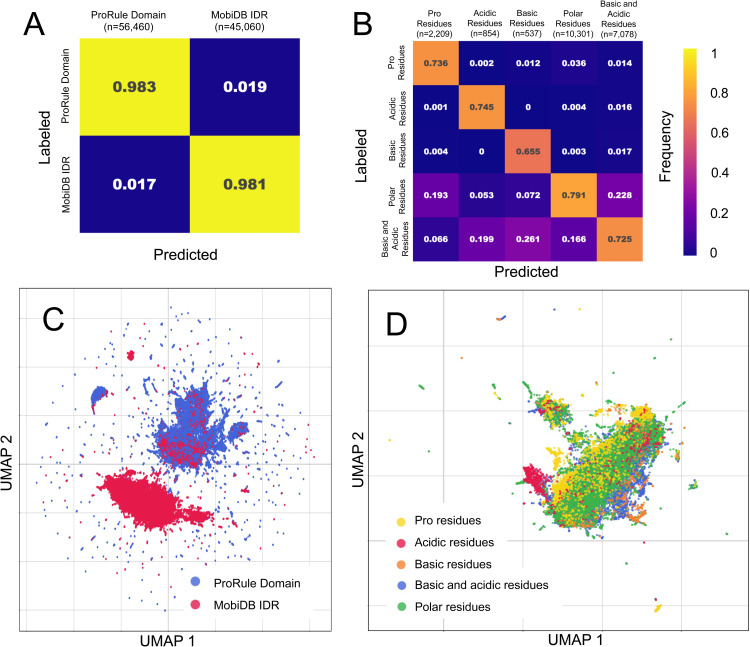
Segment Embedding Evaluation and Visualization of IDRs, Domains, and Compositional Biases. (A, B) Normalized confusion matrices for 1-nn assessment for (A) ProRule Domain compared to MobiDB IDRs (Disorder Consensus Predictions) and (B) Compositional Biases from MobiDB. In the normalized confusion matrix, we report 1-nn precision along the diagonal and state the number of ZPS segments for each label as n (see S2 Table for confidence intervals). (C, D) Shows 2-dimensional UMAPs of segment embeddings labelled with (C) ProRule domains compared to MobiDB disorder consensus and (D) Compositional Biases from MobiDB. Each point in the UMAP is a protein segment, and each segment shown in the UMAP was used in the respective 1-nn assessment.

Next, we took a closer look at different biological properties associated with function, such as whether the protein segment can fold independently and compositional biases associated with IDRs. Here we performed an evaluation where each segment can only have one label of many possible labels (multi-class classification), we selected the label with the highest IoU (see Methods). We find that MobiDB IDRs and ProRule domains are clearly separated in the segment embedding space. The 1-nn precision for ProRule domains and MobiDB IDRs is 0.983 + /-0.001 and 0.981 + /-0.001, respectively (Fig 3A and S2 Table). This is as expected since IDRs and domains can be distinguished based on amino acid composition, which we confirmed using one hot encoding (1-mer) embeddings of these segments (S2 Table). Similarly, we found compositional bias predictions from MobiDB can be distinguished by segment embeddings, where the 1-nn average precision is 0.731 + /- 0.009 (Fig 3B and S2 Table). Since these predictions are provided by MobiDB, a database with a strong focus on IDRs, this set of compositional biases are most relevant for IDRs but do not necessarily coincide with IDRs. By reducing the dimensionality of the segment embeddings using a UMAP, we can visualize segment embeddings in a scatter plot. This shows that folded domains and IDRs are more separable than compositional biases (Fig 3C and 3D). The compositional biases visualization shows more overlap between protein segments with different compositional biases, but still reveals patterns associated with their compositional bias (Fig 3D). This demonstrates that segment embeddings contain sufficient information to categorize structural state and compositional biases.

### Segment embeddings can distinguish different types of protein domains and sub-domains

**Fig 4:**
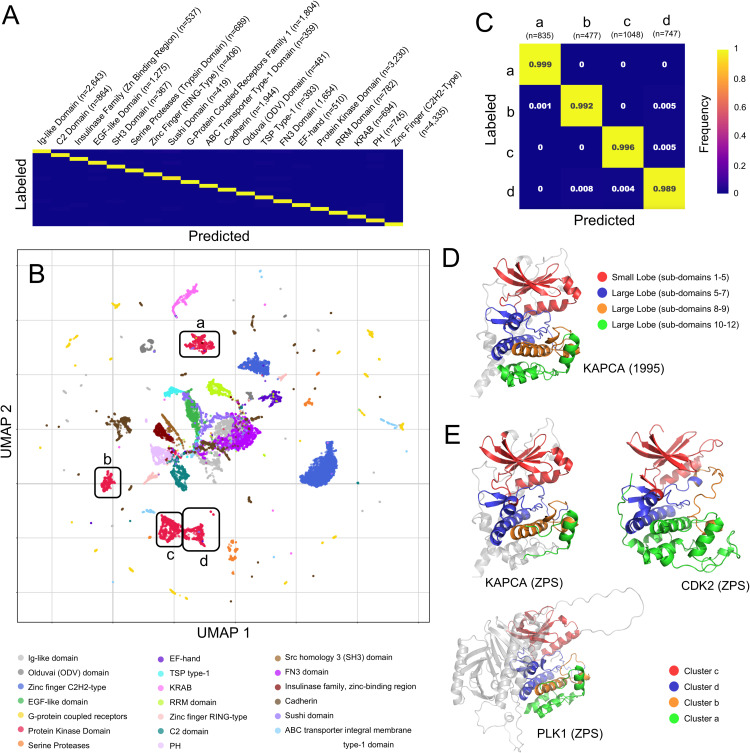
Segment Embedding Evaluation and Visualization Domain Types and Sub-Domains. (A, C) Normalized confusion matrices for 1-nn assessment, precision is shown along the diagonal, and n is the number of ZPS segments. (A) Shows the top 20 most commonly occurring ProRule domains in the ZPS segments and (C) shows clusters a, b, c, and d of the protein kinase domain (as labelled in panel B). (B) A 2-dimensional UMAP of segment embeddings coloured by the domain the segment overlaps with. Clusters a, b, c, and d of the protein kinase domain segments are defined by their position in the UMAP. Each of the protein segments shown here were used in the 1-nn assessment. (D) The AlphaFoldDB structure of KAPCA (UniProt ID: P17612) with the protein kinase domain shown in colour, with sub-domains 1-5 (red), 5-7 (blue), 8-9 (orange), and 10-12(green). Sub-domains 1-5 are the small lobe and 5-12 are the large lobe, as defined in [42]. (E) AlphaFoldDB structures of KAPCA, CDK2 (UniProt ID: P24941), and PLK1 (UniProt ID: P53350), where colours are defined by clusters of protein kinase segments, including cluster c (red), cluster d (blue), cluster b (orange), and cluster a (green).

We next tested whether segment embeddings can distinguish different types of protein domains. Here, we transfer ProRule annotations from UniProt to segment embeddings and analyze the top 20 most common domain annotations amongst the segment embeddings with a multi-class 1-nn assessment. We find different types of domains are easily distinguishable using segment embeddings and have 1-nn precisions ranging from 0.951 to 1 (+/- 0.004 to ~0) and an average precision of 0.986 + /- 0.002 (Fig 4A and S2 Table). 3-mer embeddings have a broader range of 1-nn precisions from 0.483 + /-0.010 to 0.932 + /-0.005 with an average precision of 0.762 + /-0.008 (S2 Table).

We visualized segment embeddings that overlap with a domain annotation using UMAP (Fig 4B). We observe that segment embeddings with different domain annotations form distinct clusters as a direct result of the dimensionality reduction, we did not perform any clustering analyses on the segment embeddings. Additionally, we noted some segment embeddings with the same domain annotation organized into multiple clusters, such as the protein kinase domain (Fig 4B and S3 Table). First, we established that the multiple protein kinase domain clusters exist in the high dimensional space by performing the same 1-nn assessment showing an average 1-nn precision of 0.994 + /- 0.004 (Fig 4C and S2 Table) compared to 3-mer embeddings with 1-nn average precision of 0.888 + /-0.017 (S2 Table). We then found the segments in these clusters can be ordered linearly within the protein kinase domain amino acid sequence, starting from the N-term with cluster c, then d, then b, and cluster a, for 425/481 (88%) protein kinase domains in the human proteome. From this, we determined that segments in clusters a, b, c, and d are associated with the 12 sub-domains defined in [42] as conserved regions that are uninterrupted by large insertions across an alignment of the eukaryotic protein kinase superfamily. These 12 sub-domains can be grouped into 2 lobes where each of the sub-domains and lobes have distinct amino acid conservation constraints and functional roles [42]. The small N-terminal lobe contains sub-domains 1–5 (Fig 4D, red) and the large C-terminal lobe contains domains 5–11 (Fig 4D, blue, orange and green), where sub-domain 5 spans both lobes [42]. We found that the small lobe (Fig 4D, red) corresponds to segments from cluster c (Fig 4E, red). In the large lobe, sub-domains 5, 6A, 6B, and 7, (Fig 4D, blue) identified by their structural components (a-helix D, a-helix E, and B-strands 6–9 [42]), correspond to cluster d (Fig 4E, blue). We observe slight variations with the sub-domains included in clusters a and b, most notably whether cluster a includes a few extra alpha helices (Fig 4E, CDK2) or is missing a few alpha helices (Fig 4E, KAPCA). Despite these small variations in how a few short alpha helices are distributed between clusters a and b, our approach can zero-shot segment protein domains and sub-domains and distinguish them more effectively than 3-mer embeddings.

### Segment embeddings can distinguish intrinsically disordered regions

We next assessed whether segment embeddings can be used to distinguish different IDRs. This is a difficult problem for which few supervised approaches have been developed [7,10,11]. We used 3 different sources of IDR annotations including functional IDR annotations from Disprot [43], protein localization annotations from UniProt [27] and CD-CODE [44] as seen in ProtGPS [45], and UniProt annotations that overlap with MobiDB IDR annotations. We included multi-label 1-nn analyses using Disprot and the annotations used by ProtGPS, including CD-CODE and selected UniProt annotations, because they are curated from experimental evidence. We also included a multi-class 1-nn assessment using a broader selection of UniProt annotations that range from expert curated to bioinformatics tool predictions because there are only a limited number of evidence-based IDR annotations.

**Fig 5:**
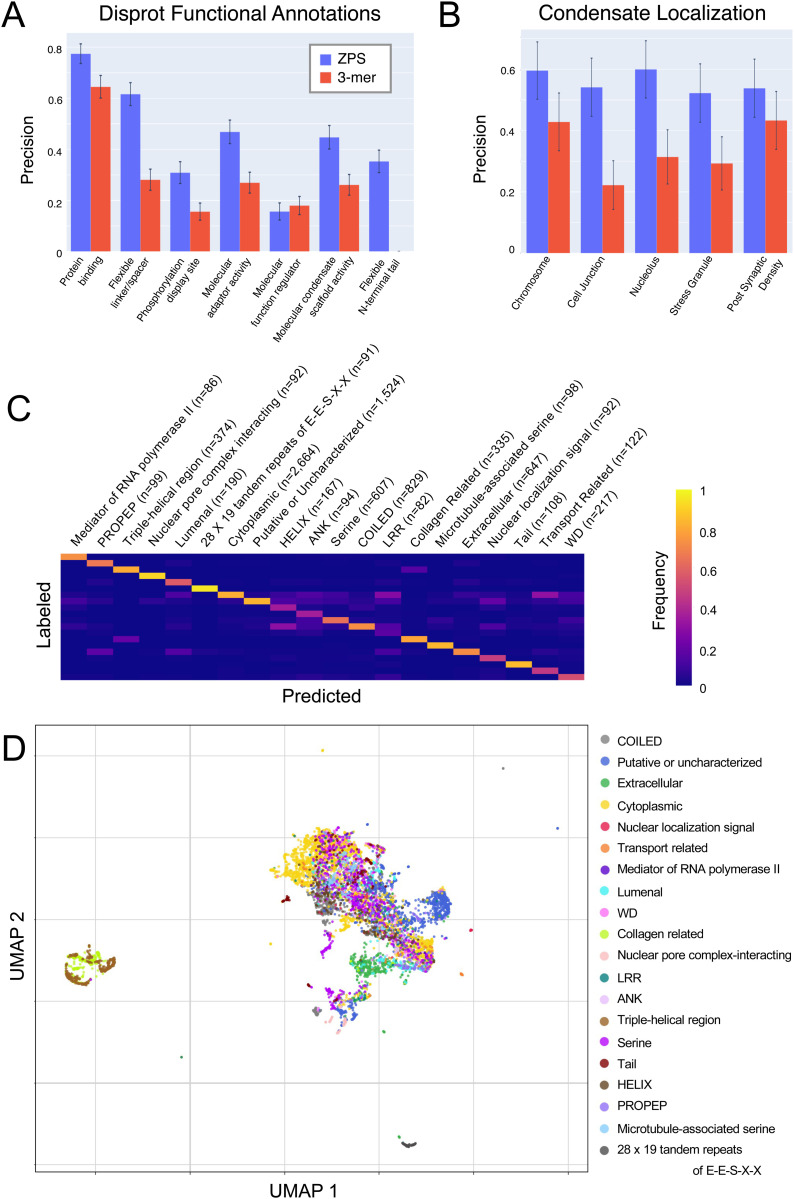
Segment Embedding Evaluation and Visualization of IDRs. (A) 1-nn precision for Disprot functional annotations, reported for ZPS (blue) and 3-mers (red). (B) 1-nn precision for ProtGPS localization annotations, reported for ZPS (blue) and 3-mers (red). Error bars represent the binomial confidence intervals and can be found along with precision values in S1 Table. (C) The normalized confusion matrix for 1-nn assessment of the top 20 most common annotations that overlap with MobiDB IDRs. Precision is shown along the diagonal, and n is the number of ZPS segments. (D) UMAP of the segments used in the 1-nn assessment labelled with the annotations that overlap MobiDB IDRs.

To test whether segment embeddings are predictive of IDR functions, we used function-related annotations from Disprot (see Methods). We found that ZPS out-performs 3-mers with an average precision of 0.446 + /- 0.042 compared to 0.256 + /-0.034, respectively (Fig 5A and S1 Table). We do not compare to other methods that predict Disprot annotations because these methods make predictions for whole proteins and are often evaluated at the residue-level [7,10,11] while ZPS makes predictions about protein segments and is evaluated at the segment-level, so the results are not directly comparable.

ProtGPS is a supervised language model that predicts the compartmental localization of whole proteins. We transfer whole protein annotations from their training, dev, and testing sets to the MobiDB IDR segments of reviewed human proteins then evaluate using a filtered set of their testing set (see Methods). We report evaluations for 5 localization classes that have sufficient testing data, where ZPS consistently out-performs 3-mers with an average precision of 0.560 + /-0.094 compared to 0.338 + /-0.089, respectively (Fig 5A and S1 Table). These results are also not directly comparable to other current methods because ProtGPS makes predictions at the whole protein level while we limit ZPS to only use MobiDB IDR protein segments.

Last, we selected all protein segments that overlap with IDR annotations from MobiDB then transferred additional overlapping annotations from UniProt and then selected the top 20 most common IDR annotations. This includes some annotations that are associated with structured domains such as collagen, WD, and HELIX. We proceeded with this set of annotations despite this contradiction to stay consistent with our use of UniProt annotations even though MobiDB disorder annotations are based on predictions that may not represent bona fide IDRs. Furthermore, there is a lack of consensus on IDR types and identifying a large and diverse set of IDR annotations remains challenging. We calculated the 1-nn precision and visualized the segment embeddings of the IDRs using UMAP (Fig 5C and 5D). We observe a wide range of 1-nn precisions with an average of 0.660 + /-0.014 (S2 Table), which we believe is due, at least in part, to the broader definition of IDR annotations compared to folded domain annotations. For example, we have labels with non-exclusive meanings such as Collagen-Related and Triple Helical Region as well as COILED and HELIX [46,47]. Nevertheless, we see a clear improvement over 3-mer embeddings which have an average precision of 0.266 + /-0.012 (S2 Table).

These results show that ZPS segment embedding similarity is more predictive than 3-mers for expert curated experimental evidence-based IDR annotations from Disprot and CD-CODE as well as UniProt annotations that overlap with MobiDB IDRs. However, we acknowledge the limitations of these experiments in the number and variety of IDR annotations that are available and investigate how ZPS can be used to identify IDRs with similar functions in the following sections.

### Discovery of unannotated mitochondrial targeting signals in the human proteome

**Fig 6:**
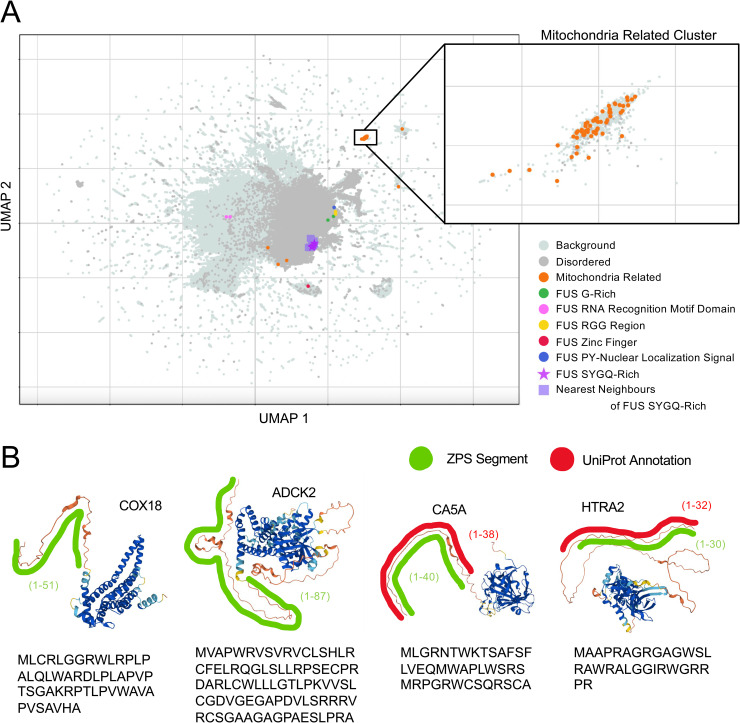
Identification of Mitochondrion Related Cluster and SYQG-Rich Prion-Like Domains Similar to FUS. (A) UMAP of segment embeddings of the entire human proteome. Disordered segments (defined by MobiDB) are shown in a warmer shade of grey to give context to the UMAP. Mitochondria Related cluster (orange) was defined by Leiden clustering of unannotated protein segments (see Methods). Segment embeddings of FUS are shown along with the top 10 nearest-neighbours to the segment embeddings of FUS’s SYGQ-rich regions. The rest of the human proteome is shown in light grey. (B) AlphaFoldDB structures and amino acid sequences of COX18 (UniProt ID: Q8N8Q8, amino acids 1-51), ADCK2 (UniProt ID: Q7Z695, amino acids 1-87), CA5A (UniProt ID: P35218, amino acid 1-40), and HTRA2 (UniProt ID: O43464, amino acids 1-30) colored by pLDDT confidence score (very low in orange, low in yellow, high in cyan, and very high in blue), showing UniProt mitochondrion targeting signal annotations (red) and ZPS segments (green). COX18 has a UniProt mitochondrion targeting signal annotation of unknown length starting at the N-terminus and ADCK2 has no UniProt mitochondrion targeting signal annotation but is known to localize to the mitochondrion [51].

To investigate whether the segment embeddings can be used to make predictions about unannotated protein segments, we used Leiden clustering [48,49] to identify clusters of unannotated segments in the human proteome (see Methods). One of these clusters is shown in the context of the whole human proteome using a UMAP (Fig 6A, orange). After correcting for over-segmentation (See Methods) there are 51 unannotated segments in this cluster that are separate from the majority of the human proteome (Fig 6A, orange, and S4 Table). We found that there are 609 annotated segments that appear near this cluster on the UMAP (Fig 6A, light grey, Mitochondria Related Cluster, and S4 Table), which overlap with 435/495 (87.8%) of the annotated mitochondrion targeting signals in the human proteome from UniProt. Consistent with this, since typical mitochondrial targeting signals are located at the N-terminus of the protein sequence [50], 644/660 (97.6%) of segments in the Mitochondria Related Cluster, including both annotated (Fig 6A, light grey, Mitochondria Related Cluster) and unannotated (Fig 6A, orange, Mitochondria Related Cluster) begin at the N-terminus of the protein. Further, 625/648 (96.5%, 7 unmapped proteins) of the proteins containing these segments localize to the mitochondria (p = 0), including 44/48 (91.7%, 3 unmapped proteins) proteins with unannotated segments (p = 8.98E-39). Interestingly, UniProt includes 58 mitochondrion targeting signals of unknown length in the human proteome that begin at the N-terminus of the protein, of which 39 (76.5%) are found in this cluster of 51 unannotated segments. This evidence strongly suggests that the remaining 12 unannotated segments in this cluster (Fig 6A, orange) are undiscovered mitochondrion targeting signals, and we can use the boundary predictions of ZPS to determine the size of the 39 mitochondrion targeting signals of unknown length.

To test whether segment embeddings associated with this unannotated segment cluster (Fig 6A, orange) are simply the N-terminal IDRs of mitochondrial proteins instead of actual mitochondrion targeting signals, we compared examples of these segments to predicted structures from AlphaFoldDB and annotated mitochondrial targeting signals from UniProt. In some cases, such as CA5A, the boundaries of UniProt annotations of mitochondrial targeting signals (Fig 6B, red) match closely with ZPS boundaries (Fig 6B green) and a region having AlphaFold pLDDT confidence scores <50 (“very low”, highly correlated with structural disorder, Fig 6B, orange). However, in other cases such as HTRA2, the UniProt mitochondrion targeting signal occupies only the first 30 residues (Fig 6B, red) with the ZPS boundaries matching the UniProt annotation (Fig 6B, green), while the very low confidence region spans the first 131 residues (Fig 6B, orange). This use of ZPS and segment embeddings to annotate mitochondrion localization signals provides evidence that pLM embeddings can provide valuable insight without supervised training or fine-tuning.

### Identifying similar IDRs to FUS’s SYGQ-rich region using the k-nearest neighbours of segment embeddings

We chose to investigate the 4 SYGQ-rich segments in FUS because these segments represent “prion-like domain” elements of IDRs with well-studied role in forming condensates, such as in response DNA damage stress, or in forming aggregates, such as in amyotrophic lateral sclerosis and frontotemporal dementia [52–54]. In general, prion-like domains have a strong compositional bias and can be identified from amino acid sequences using Prion-Like Amino Acid Composition (PLAAC) scores [55].

**Fig 7:**
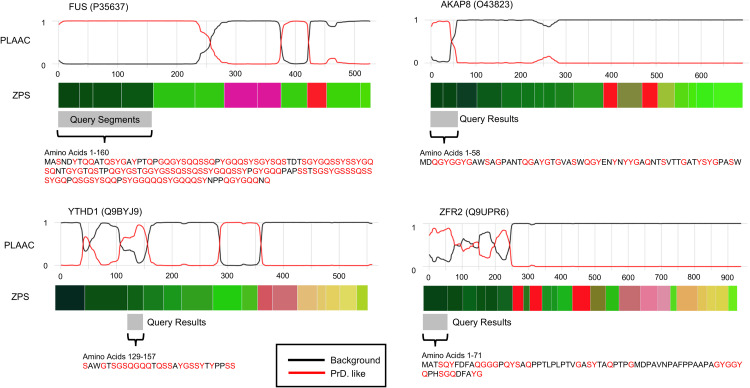
Zero-Shot Protein Segmentation of Proteins with Similar Segments to FUS’s SYGQ-Rich Region and PLAAC scores. Prion-Like Amino Acid Content (PLAAC) scores adapted from the web tool described in [55], segment embedding visualization (ZPS) as shown in Figs 1 and 2, and the amino acid sequence of the query segments from FUS and query result segments (S, Y, G, and Q shown in red).

We identified the 10 nearest-neighbours for each of the 4 segments of FUS’s SYGQ-rich prion-like domain in the human proteome. After removing duplicates and joining adjacent segments (see Methods), we have 14 segments from 11 proteins (S5 Table). On average the amino acid content of these segments is 56% SYGQ, and 771/943 (81.8%) amino acids are predicted to be in a prion-like domain by PLAAC (Fig 7 shows 4 examples). Encouragingly, the SYGQ-rich regions of all 3 FET proteins (FUS, EWSR1, and TAF-15) were included in these segments, given the known conservation of these prion-like domains across the FET family [30,56,57]. Despite visually similar sequence composition in these amino acid sequences (Fig 7), performing a protein BLAST [58] using both default and low complexity specific parameters, on the human proteome using FUS’s SYGQ-rich prion-like domain only retrieves anomalous fusion proteins, isoforms of FUS and EWSR1, and a few unnamed protein sequences – the search does not identify any other reviewed human proteins.

Interestingly, in addition to the FET family, we identified 8 other proteins with similar segment embeddings to FUS’s SYGQ-rich regions. Encouragingly, the segments in 3 of the 8 proteins, YTHD1, YTHD3, and AKAP8, have been reported to form condensates and bind RNA, just as the FET family’s SYGQ-rich regions can. Similar to FUS, the N-terminal IDR in YTHD1 and YTHD3 is required to form condensates with RNA in the cytosol in response to stress [59,60]. Also, similar to FUS, AKAP8 forms condensates in the nucleus and binds RNA to regulate splicing, and condensate formation is required for RNA regulation [61]. While other elements of AKAP8 have been identified to be required for condensation [61], we predict that the prion-like domain segment (Fig 7) makes a significant contribution to condensation. We observed other prion-like domains predicted by PLAAC that are not nearest-neighbours of FUS (Fig 7, bright green segments). However, in FUS these segments have a different compositional bias, G-rich or RGG (which also has high G content), that is also known to be enriched in prion-like domains [55]. This suggests we can distinguish prion-like domains by their compositional biases, beyond the binary classification provided by PLAAC. Taken together, our analysis suggests that the most similar segment embeddings to those of FUS’s SYGQ-rich prion-like domain are other prion-like domains with similar compositional biases that cannot be found using BLAST or uniquely identified by PLAAC.

## Discussion

Here we show that embeddings from ProtT5 can be used to predict the boundaries of protein regions and categorize protein segments without training or fine-tuning any parameters. We reproduced the boundaries of annotations from UniProt more effectively than established bioinformatics tools and demonstrated that segment embeddings are more informative than k-mer embeddings at categorizing UniProt annotations (n>=25), including protein domains, sub-domains, and types of IDRs. We then used these approaches to identify unannotated mitochondria localization signals and SYGQ-rich prion-like domains in the human proteome. Additionally, we present a novel approach for visualizing pLM embeddings using protein segments and colours.

To visualize the high-dimensional segment embeddings, we represented the segments as colours along the protein sequence length. Colouring proteins by distinct annotations is widely done in the molecular biology literature to help understand complex proteins [29,30,32,34]. We found that for well-characterized RNA-binding proteins, we could reproduce these diagrams using boundaries defined by ZPS and colours resulting from a dimensionality reduction on segment embeddings. We believe that these visualizations will be a useful tool for biologists to visualize and compare the functional organization of proteins that they study. Further, we believe that converting embeddings from unsupervised language models to RGB-colour space represents a new way to visualize the information learned during pre-training that can be applied to any language model (Ami G. Sangster, Micaela Consens, Alan M. Moses, in preparation).

The boundaries defined by ZPS over-segment proteins relative to annotations on UniProt and the literature (Figs 1–2 and 4). While this leads to poorer performance when benchmarking against annotations from UniProt, when we investigated well-annotated proteins, we found that the over-segmentation captured meaningful biology such as motifs in structured domains and IDRs (Fig 1, RRM and RGG) as well as sub-domains (Fig 4). We also observe over-segmentation relative to IDR annotations where some segments align with different annotations (Fig 1, C-term RGG and PY-NLS), while other instances of over-segmentation (Fig 1, N-term multiple SYGQ-rich segments) remain unexplained by the currently available annotations. Given the motifs and sub-domains identified by over-segmentation relative to UniProt and some literature annotations, it’s likely that this “over-segmentation” also represents a more detailed and informative annotation.

Evaluating ZPS as a segmentation problem challenges conventional ideas about standard evaluations metrics used for bioinformatics problems. For example, segmentation approaches take one input, a sequence, and provide multiple outputs, segments, for which there are many possibilities. This impacts measures such as the precision-recall curves because none of these methods predict all possible segments, so recall never goes to one even when precision nears zero (S2 Fig). This puts more importance on having high recall at a reasonable number of predictions because this means the method is effectively identifying annotated segments from a very large set of possibilities. Furthermore, with this problem in particular, the reported precision is a lower bound because over-segmentation relative to annotations makes the predictions too small to be considered a true positive and some of the predictions may correspond to annotations that are not yet included in protein databases, so these are all counted as false positives. However, we don’t anticipate that having a poorer precision on the segmentation task will have negative impacts on ZPS downstream utility because unannotated segments and over-segmentation are two prevalent causes which can be easily remedied by analyzing the top nearest neighbours of segment embeddings for an enrichment of functional annotations or segments adjacent to the query segment. Additionally, identifying segments that are not yet included in protein annotation databases, including those identified by over-segmentation, is one of the advantages of using ZPS.

ZPS demonstrates an advantage over classical bioinformatics approaches, such as HMMs and alignment-based methods [2,58,62], because ZPS does not rely on positional evolutionary conservation of primary amino acid sequence. Consistent with this, ZPS can identify the boundaries of IDRs and regions with compositional biases (Tables 1 and 2), which have poor positional conservation at the primary amino acid level but have meaningful conservation of amino acid properties distributed across their sequence [8,9]. Furthermore, these types of protein segments can be categorized using segment embeddings (Figs 2–3 and 5). We can also use segment embeddings to identify IDR protein segments that are believed to have similar functions, such as mitochondrial targeting signals and SYGQ-rich prion-like domains that drive condensation. While we believe that segment embeddings contain information about sequence characteristics and organizational paradigms that are important for the functional classification of IDRs, identifying these remains an area for future work since interpreting the embedding space of pLMs remains an area of active research [63,64].

Additionally, unlike other deep learning methods that use supervised learning, fine-tuning, or transfer learning, our approach is zero-shot. Generalizability is therefore less impacted by the content or availability of training data. This enables us to identify protein regions for which there are too few known examples to train a supervised deep learning model, such as SYGQ-rich prion-like domains. In principle, this means we can discover novel categorizations of protein regions that do not have any current characterized examples. Practically, this means ZPS can be used to generate hypotheses about the functions of protein segments for which there are no current tools. Additionally, training or task-specific fine-tuning on segment embeddings would likely lead to improved performance for identifying boundaries and categorizations of known regions for tasks with sufficient training data. This has been demonstrated recently for genome language models that were fine-tuned for genome segmentation [65].

ZPS has some limitations. First, as is widely appreciated, the pLM embedding space is not interpretable. This means that ZPS does not provide insight into what aspects of the protein sequence are important for the predictions. Future work could apply interpretation techniques for Language Models [63,64] to ZPS. Second, during the course of our research, we noted that less-appreciated inference-time batch and hardware dependent behaviours of pLMs -can impact ZPS. Since protein sequences are gathered into batches at inference time, we find that the embedding of each protein is slightly influenced by the other proteins in the batch. Additionally, since pLM embeddings depend on billions of floating-point operations, the exact numerical values obtained in embeddings may be hardware dependent. We found that the combined effects of these batch and hardware dependencies affect around 1% of ZPS boundary predictions, where most often the boundary is shifted by a few amino acids. Last, ZPS is based on ProtT5 which requires the use of a GPU with substantial VRAM, which may not be available to all researchers. In our hands, one 16GB GPU was sufficient to segment the human proteome. To increase access for researchers, we have made a google colab notebook available that can segment small numbers of proteins.

Looking forward, we anticipate that the continuing developments in image segmentation and deep learning can be applied to make further advancements in protein annotation and genomics as well as their evaluation. Our work is a first step towards integrating the advancements of zero-shot image segmentation and protein language models for understanding functional organization within proteins.

## Methods

### RNA-binding protein annotations from the literature

Annotations from the literature are shown in Figs 1 and 2 for RNA binding proteins, including FUS (UniProt ID: P35637), EWSR1 (UniProt ID: Q01844), TAF-15 (UniProt ID: Q92804), hnRNPA1 (UniProt ID: P09651), and TDP-43 (UniProt ID: Q13148). We show annotations along the protein’s sequence as described in the literature [29,30,34].

### UniProt protein sequences and annotation data

For the segmentation evaluation, we use protein sequences and annotations for the human proteome from UniProtKB/Swiss-Prot (UniProt) [27] (available on Zenodo). The sequences on UniProt have reviewed annotations from a variety of sources including bioinformatics tools, academic literature, and other databases. Here “annotations” only refer to annotations that are associated with specific amino acids in the protein (available on Zenodo). We removed annotations that include non-numeric characters in their sequence positions, such as “1-?”, “100-?150” and “<1-100”, as it indicated uncertainty in the position or length of the sequence in the annotation. For evaluating protein segmentation, we use annotations from UniProt with any evidence level, including those from sequence analysis, prediction, curation, citation, MobiDB [28], and ProRule, a database provided by Prosite [2]. To assess performance on specific types of protein segments, we include a partition of UniProt that only includes IDR consensus predictions and compositional bias annotations from MobiDB. Additionally, we use a partition of UniProt that only includes domains annotated by ProRule.

### Zero-shot protein segmentation (ZPS)

We use the ProtT5-XL-UniRef50 [14] encoder at half precision (available on hugging face at Rostlab/prot_t5_xl_half_uniref50-enc) to generate protein embeddings for the whole human proteome for sequences less than 8k amino acids in length. Here we use “embedding” to refer to the output of the ProtT5 encoder, sometimes also referred to as a “representation”. The ProtT5 embedding is a 2-dimensional matrix where one dimension is the length of the protein and the other is the embedding space of length 1024 (Lx1024). Next, a change point analysis is performed on each ProtT5 embedding (see below for Change Point Analysis). This defines the boundaries between protein segments. For example, if the boundaries are a, b, and c in a protein of length 100, the segments are (0, a), (a, b), (b, c), and (c, 100). These ranges include the first position and go up to but not including the second, as conventionally seen in zero-based indexing. These segments are defined without fine-tuning or training any parameters. See S1 Fig for a visualization of ZPS and defining ZPS colours.

### Change point analysis

A change point analysis is commonly used to detect changes in a signal or time series [66]. We selected this method to perform zero-shot protein segmentation because it does not require training parameters. We use a sliding window with a window size of 30 for our search algorithm and RBF kernel as our cost function. We chose a sliding window algorithm because this has previously been used in protein biology to identify modular organization within proteins [13]. This compares a segment of 15 amino acids to the next 15 amino acids for all amino acids in the protein. If there is a significant change between the first 15 amino acids and the next 15 amino acids, the space between them becomes a boundary that defines protein segments. See [66] for sliding window algorithm and RBF kernel function definition.

The number of boundaries predicted by the change point analysis is determined by one of two pre-selected hyperparameters: either set a threshold on the cost function that acts across the whole dataset or set a threshold on the number of boundaries for each protein. Interestingly, we found a way to avoid doing either, making our approach free from hyper-parameter selection in addition to not training or fine-tuning any parameters. Initially we set a threshold on the number of boundaries per protein based on the protein’s length, but we found that allowing more than 3 boundaries per 100 amino acids did not increase the total number of predicted boundaries. Therefore allowing 3 or more boundaries per 100 amino acids allows the change point analysis to predict the maximum number of boundaries it can find. To allow the method to function without restraint, we gave the threshold of 3 boundaries per 100 amino acids, which yields approximately 253k segments. Compared to unsupervised methods we benchmark against, such as fLPS2 (109k segments) and Chi-Score Analysis (554k segments), 253k segments is reasonable considering fLPS2 is tailored towards a single type of protein segment whereas ZPS and Chi-Score are attempting to predict any type of protein segment, so we moved forward with this set of predictions.

### Segment embeddings

We define segment embeddings by cutting the residue level whole protein embedding at the boundaries defined by the change point analysis and then average pooling the segmented embedding to 1x1024. For example, if a segment spans amino acids (100, 125) the ProtT5 protein embedding would be cut at position 100 and 125 producing an embedding of size 25x1024 which would be average pooled down to 1x1024. Segment embeddings were generated without training or fine-tuning any parameters. We did not generate segment embeddings by passing protein fragments to ProtT5.

### K-mer embeddings

We use k-mer embeddings as a baseline comparison for the content of the segment embeddings. K-mer embeddings are defined using the same protein segments as segment embeddings defined by ZPS. 1-mer embeddings are equivalent to one hot encoding where each dimension in the embedding represents the number of occurrences of each amino acid. 3-mer embeddings contain the number of occurrences of each permutation of 3 amino acids (AAA, AAC, AAD, …). We use overlapping k-mers and normalize k-mer embeddings before evaluations.

### Correcting for over-segmentation

After analyzing FUS and other RNA-binding proteins, it was apparent that ZPS over-segments relative to annotations on UniProt despite the meaningful biology, such as motifs, that is captured by this over-segmentation (Figs 1 and 2). We attempt to correct for over-segmentation by merging adjacent segments if they have similar segment embeddings. We measure the cosine similarity between all segments in a protein; if the segments with the highest cosine similarity are adjacent to each other in the protein sequence, then they are merged. We only correct for over-segmentation if there are 6 or more segments in a protein since this algorithm will join all segments in a protein if it has a small number of segments. For example, if there are only 3 segments in the protein, then the middle segment will always get merged since it is adjacent to all the other segments of the protein, regardless of their similarity. Increasing the number of segments to 6 reduces the chance of “accidental” merging while still reducing the total number of segments by ~100k (40%).

We also correct for over-segmentation when analyzing clusters (Figs 4 and 6). Here, we simply join adjacent protein segments that are in the same cluster.

### UMAPs

We used Uniform Manifold Approximation and Projection (UMAP) [67] to reduce the dimensionality of segment embeddings in two different instances. One is to visualize segment embeddings in a 2-dimensional scatter plot and the other is to visualize segment embeddings as 3-dimensional colours (see next section).

### Segment embeddings to RGB colours

For our visualization approach, we reduced the segment embeddings (1x1024) of the whole human proteome to 3 dimensions (1x3) using a UMAP [67]. First, each of the 3 dimensions is mean centered across the whole proteome, then we apply a sigmoid function to each of the RGB values for all segments and multiply by 255. We do this to scale the values and change their distribution from an approximately normal distribution to a more uniform distribution. This shifts the colours from mid-tone greys to increase their vibrancy and make the colours more visually distinct from each other. After this step, each segment has 3 values ranging from 0-225, one for red, green, and blue, which can be directly converted to a specific RGB colour. See S1 Fig for a visualization of sequence to ZPS colours.


$$X=[X_{\text{1}},X_{\text{2}},X_{\text{3}}]=\text{UMAP}(\text{human}\;\text{proteome}\;\text{segment}\;\text{embeddings})$$

$$[R,G,B]=\frac{\text{2}\text{5}\text{5}}{\text{1}+e^{-(X-mean(X))}}$$

This function is applied elementwise, where X_1_ maps to R, X_2_ maps to G, and where X_3_ maps to B.

### Arginine clustering and bootstrap analysis

Arginine embeddings were clustered by hierarchical agglomerative clustering, and we used a threshold of 0.5 to define different clusters. Cluster 1 is a cluster of small outlier clusters. We use a bootstrap with n = 10,000 to measure the chance of observing the percentage of methylation sites in cluster 2.

### Supervised bioinformatics approaches for protein segmentation

To our knowledge, there are not any supervised bioinformatics approaches designed for segmenting proteins that do not focus only on a specific type of protein region, such as domains. So, we use two supervised bioinformatics approaches that are designed to identify and categorize protein domains, Pfam and Prosite. We use HMMER 3.1b2 [62] to identify protein domains defined by Pfam (Pfam-A.hmm) [1] with default parameters. We use Pfam annotations with an e-value less than or equal to 0.01. Additionally, we define another set of domains with Prosite Scan from ps_scan_linux_x86_elf.tar.gz (updated 2018) also using default parameters [2]. We use Prosite Scan annotations if they have a corresponding normalized profile score, which can be interpreted as a confidence score. Prosite Scan is not to be confused with ProRule. While both are provided by PROSITE, Prosite Scan is an automatic domain annotation tool and ProRule is more of a “master set” of domain annotations including domains defined by manual curation. ProRule is included in UniProt.

Since both Prosite Scan and Pfam can make multiple overlapping and identical predictions (with different labels), and since labels are ignored in our segmentation evaluation, we filtered out predictions with a lower score for Prosite Scan or a higher e-value for Pfam if they have an IoU > 0.5 (see Below for IoU).

### Unsupervised bioinformatics approaches for protein segmentation

We use unsupervised bioinformatics approaches designed to segment proteins according to changes in amino acid compositional biases as a contrast to domain annotation tools. We ran fLPS2 [12] on the whole human proteome with default parameters and parameters specifically for short low complexity compositional biases (./fLPS2 -t1e-5 -m5 -M25, see fLPS2 README for further details). fLPS2 predicts protein segments with compositional biases and can make multiple overlapping predictions, we also filtered out overlapping predictions with a lower score if they have an IoU > 0.5 with another prediction. We also ran a Chi-Score Analysis [13], which predicts boundaries where there are significant changes in amino acid composition and has a built-in boundary filtering approach. We evaluated this approach with and without filtering boundaries. For Chi-Score Analysis we only included sequences less than 2k in length due to time complexity issues.

### Intersection over Union (IoU)

The Intersection over Union (IoU) is the number of amino acids in the intersection of a pair of predicted and annotated segments divided by the number of amino acids in the union of those two segments. This produces a measurement that is relative to the size of both segments. Predicted segments include those defined by ZPS, Pfam, Prosite Scan, fLPS2, and the Chi-Score Analysis. Annotated (or positive) segments include human protein annotations from UniProt that are at least 30 amino acids in length and do not span the entire length of the protein from proteins that are at least 60 amino acids in length.


$$\begin{array}{c} \text{IoU}=\frac{Predicted\;\cap \;Annotated}{Predicted\;\cup \;Annotated} \end{array}$$

$$\begin{array}{c} \text{IoU}=\frac{\text{max}[\text{min}\left({Predicted}_{end}{,\;Annotated}_{end}\right)-\text{max}\left({Predicted}_{start}{,\;Annotated}_{start}\right),\text{0}]\;}{\text{max}\left({Predicted}_{end}{,\;Annotated}_{end}\right)-\text{min}\left({Predicted}_{start}{,\;Annotated}_{start}\right)} \end{array}$$

Next, we measure the average IoU. Here we show the average IoU for the positive (pos) and predicted (pred) segments. Conventionally in image segmentation “average IoU” refers to the average IoU of the predicted segments, meaning for each predicted segment we take the top IoU with any positive segment then average those values. The average IoU of the positive segments means that for each positive segment we take the top IoU with any predicted segment, then average those values. We chose to report both in this analysis because, surprisingly, they are greatly different (Table 1) and offer additional insights to the model’s performance.

We use a threshold of 0.5 on IoU to define precision and recall. If a pair of predicted and annotated segments have an IoU above the threshold, it is a true positive. If a predicted segment does not have a pair above the threshold, it is a false positive. If an annotated segment does not have a pair above the threshold, it is a false negative.


$$Recall=\frac{True\;Positives}{True\;Positives+False\;Negatives}$$

$$Precision=\frac{True\;Positives}{True\;Positives+False\;Positives}$$

Here, precision should be considered a lower bound, since there are likely many functional segments that are not yet annotated in protein databases.

### Precision-recall curves

To plot the precision-recall curves (S2 Fig) we calculated a normalized score to rank the confidence of the predictions made by each method; Pfam (e-values), Prosite Scan (profile scores), fLPS2 (p-values), and chi-score (score). For ZPS we calculated a score for each boundary by performing a t-test to compare the 15 amino acids before a boundary to the 15 amino acids after the boundary, similar to sliding window of the change point analysis. The score for each segment is the average score of the two boundaries that define it. Segments on either end of the protein only have one boundary with a score (the boundaries at beginning and end of the protein do not have scores), so that score was used as the score for the whole segment.

We manually calculated and plotted precision and recall at k predictions because many of the scores we used (p-values, e-values, etc.) have a heavily tailed distribution and did not visualize well at different score thresholds. Additionally, built-in precision-recall functions in python assume recall is 1 when precision is 0 and this is not true for this case (see Discussion).

### Boundary evaluation

To evaluate the predicted boundaries, we measure the difference in amino acids between the beginning and end of each annotated segment and the predicted segment with the highest IoU. The difference at the beginning and end are counted equally towards the percentage of boundaries within 10 amino acids. Annotated segments that do not overlap with any predictions are counted as an “unpaired segment”. Unpaired segments are also counted towards the percentage of boundaries within 10 amino acids as a boundary that is over 10 amino acids away. We include these in the total percentages so that this measure is relative to the total number of annotations. Since we count the unpaired annotations towards the total percentage, the table shows the percentage of annotated segments have a predicted boundary within 10 amino acids.

### Transferring UniProt annotations to segment embeddings for multi-class assessments

We transfer annotations from UniProt to segments defined by ZPS based on how much the segment overlaps with a UniProt annotation. When labeling segments, we allow UniProt annotations of any size, as opposed to evaluating segmentation/boundary prediction only allow annotations that are 30 amino acids or larger. We handle the many overlapping annotations that can be found on UniProt by selecting the annotation that has the highest IoU with the ZPS protein segment. An annotation is only considered to be transferred if the intersection between the UniProt annotation and the ZPS protein segment is at least 30% of the ZPS protein segment. This approach was applied to experiment shown in Figs 3 and 4.

When we consider annotations that are overlapping IDRs to assess segment embeddings of IDRs (Fig 5C and 5D), we first define ZPS segments as IDRs if at least 30% of their amino acids overlap with a disordered annotation from MobiDB, then we perform the annotation transfer described above only for ZPS segments that are at least partially IDRs.

### Transferring multiple annotations for multi-label assessments

For multi-label assessments (S1 Table) we allowed an annotation to be transferred to a protein segment if the intersection is at least 30% of the ZPS protein segment. For the UniProt analysis we only used annotations that appear at least 25 times in the human proteome because with a lower number of annotations confidence intervals were too large.

For the Disprot analysis we used the same requirements for the intersection and number of annotations. Prior to transferring annotations to protein segments, we filter Disprot to only include function-related annotations (term_namespace = “Molecular function” or “Disorder function”). Second, we noticed that Disprot includes multiple similar annotations with the same label in the same protein, if any of these have a high enough intersection with a protein segment, the annotation is transferred.

Since annotations used to evaluate ProtGPS were made at the whole protein level and we are using these annotations to evaluation IDR function, we only transfer annotations to IDR segments. Similar to our UniProt IDR analysis, we define protein segments as IDRs if at least 30% of the segment overlaps with a MobiDB disorder prediction. Then all the ProtGPS annotations for that protein are transferred to each segment. Prior to transferring annotations to protein segments, we filtered the ProtGPS data to only include reviewed human proteins, proteins that include a MobiDB disorder annotation and removed duplicates. This reduced the number of protein annotations by over half (from 5354 to 1797) (available on Zenodo). Since the data was greatly reduced, we only report annotations for which there are at least 25 examples in the testing data, otherwise the confidence intervals are too large. Additionally, since ProtGPS is a supervised method, the training, dev, and test splits were included in their data. We only evaluated on the testing set, but we allowed segments in the training and dev sets to be in the 1-nearest neighbour assessments.

### 1 nearest neighbour (1-nn) assessments

Here we use nearest neighbour to identify similar segment embeddings using cosine distance. We use this to predict the annotations of segment embeddings by using the label(s) of their 1-nearest neighbour (1-nn) as the prediction. For the ProtGPS evaluation, we do not allow predictions to be from the same protein, since annotations are made at the whole protein level, all segments in the protein have the same annotation. All other analyses allow matches from the same protein so long as the segments are not adjacent along the sequence of the protein. In the case where the 1-nn is not allowed by these rules, the next nearest neighbour is selected.

For multi-label analyses where each segment can have multiple labels, we evaluate each label as a binary classification task and report precision in the text along with supplementary data (S1 Table). For multi-class analyses where we only allow each segment to have one label, the label of the 1-nn is the prediction and we present a normalized confusion matrix in figures along with reporting precision and supplementary data (S2 Table). The normalized confusion matrix is the confusion matrix of counts where each column is divided by the number of predictions for each label, so the diagonal shows precision. For both multi-class and multi-label assessments, we report a binomial proportion confidence interval (S1 and S2 Tables).

For all analyses except the UniProt (n>=25) we find the 1-nn by calculating the all-by-all cosine similarity matrix. The all-by-all UniProt analyses included over 150,000 segments and due to space complexity issues, we used an approximate nearest neighbour search to identify the 1-nn.

We also use nearest neighbours to find protein segments that are similar to the SYGQ-rich prion-like domain in FUS (Fig 7).

### PyMOL

We use open-source Pymol available on github. We used their interactive interface to colour protein structures by protein segments defined by ZPS (Figs 2C, 4D and 4E).

The PyMOL Molecular Graphics System, Version 2.5.0 Schrödinger, LLC.

https://github.com/schrodinger/pymol-open-source

### Clustergrams with Plotly

We use clustergrams available through Plotly DashBio application to generate heatmaps of ProtT5 embeddings, segment embeddings, and amino acid embeddings and perform clustering (Figs 1 and 2). We cluster using hierarchical agglomerative clustering with average linkage and cosine distance; we also use optimal leaf ordering.

### Clustering unannotated segments with Leiden

We use ScanPy [48] to perform Leiden clustering [49] of unannotated ZPS segments from the human proteome. We do not reduce the dimensionality of the segment embeddings before clustering, and we allow for the model to find the optimal clusters; we do not limit the number of iterations it runs and chose a cluster resolution of 2.

### GO enrichment (PANTHER)

To determine which proteins localized to the mitochondria, we performed a GO Enrichment using PantherDB [68] (Version 19.0, released 2024-06-20). We report FDR corrected p-values.

## Supporting information

S1 Table Results for the multi-label 1-nearest neighbour evaluation of UniProt (n>=25), Disprot, and ProtGPS training/dev/testing data (CD-CODE and curated annotations from UniProt). This contains separate tables within excel for ZPS and 3-mer embedding evaluations of UniProt (n>=25), Disprot IDR functional annotations, and ProtGPS data (CD-CODE and curated annotations from UniProt). The results include the true positive, true negative, false positive, and false negative counts as well as the average precision, recall, and accuracy with corresponding binomial proportion confidence intervals.
(XLS)

S2 Table Multi-class 1-nearest neighbour confusion matrix, precision, and binomial proportion confidence intervals. See separate tables within the excel file. This includes confusion matrices, precision calculations, and binomial proportion confidence intervals for IDR vs domain, 5 compositional biases, protein domains, protein sub-domains, and IDRs as well as the corresponding 1-mer (one hot encoding) or 3-mer tables, for instances when they are compared to these in the main text.
(XLS)

S3 Table Protein identifiers and segment boundaries of protein kinase domain segment clusters. Identifiers are UniProt IDs, boundaries use zero-based indexing and labels include a, b, c, and d as shown in Fig 4. These segments have not been corrected for over-segmentation, meaning there can be multiple segments from the same proteins (UniProt IDs are non-unique).
(TSV)

S4 Table Protein identifiers and segment boundaries of protein segments in the mitochondrion analysis. Identifiers are UniProt IDs, boundaries use zero-based indexing and labels include “Annotated” (only includes protein segments that overlap with Mitochondrion Related cluster in the UMAP, Fig 6A) and “10” (corresponding to Leiden cluster number 10, labelled as “Mitochondrion Related” in cyan in Fig 6). These segments have not been corrected for over-segmentation, meaning there can be multiple segments from the same proteins (UniProt IDs are non-unique).
(TSV)

S5 Table Protein identifiers and segment boundaries of protein segments similar to FUS’s SYGQ-rich prion-like domain. Identifiers are UniProt IDs and boundaries use zero-based indexing. These segments have been corrected for over-segmentation, meaning “POS” contains a list of start and stop boundaries of each segment for each protein.
(TSV)

S1 Fig Simplified depiction of the ZPS approach and deriving ZPS colours. From top to bottom, starting with a protein sequence, we pass it to the ProtT5 encoder which produces a residue-level protein embedding, where the size of the embedding is 1024 by the length of the protein. Next, we perform a change point analysis which identifies boundaries (or break points) along the proteins sequence, which define the segments of the protein. The portion of the residue-level embedding that is contained within these segments is average pooled into “segment embeddings”. Then the segment embeddings are reduced and scaled to represent RGB colours. Last, the segments defined by the change point analysis shown with the ZPS colours to visualize the segment boundaries and corresponding segment embeddings. Note, this figure was designed as a simplified depiction of what happens between panel A and B of Fig 1, it does not show the actual boundaries and ZPS colours of FUS (though they appear similar).
(TIFF)

S2 Fig Precision recall curves for segmentation evaluation of (A) UniProt and the (B) MobiDB and (C) ProRule partitions on UniProt. (TIFF)
