# Supplementary figures and images for: Zero-shot segmentation using embeddings from a protein language model identifies functional regions in the human proteome
Source: PLoS Comput Biol. 2025 Nov 11;21(11):e1012929. doi: 10.1371/journal.pcbi.1012929 (PMC12617893; doi:10.1371/journal.pcbi.1012929)

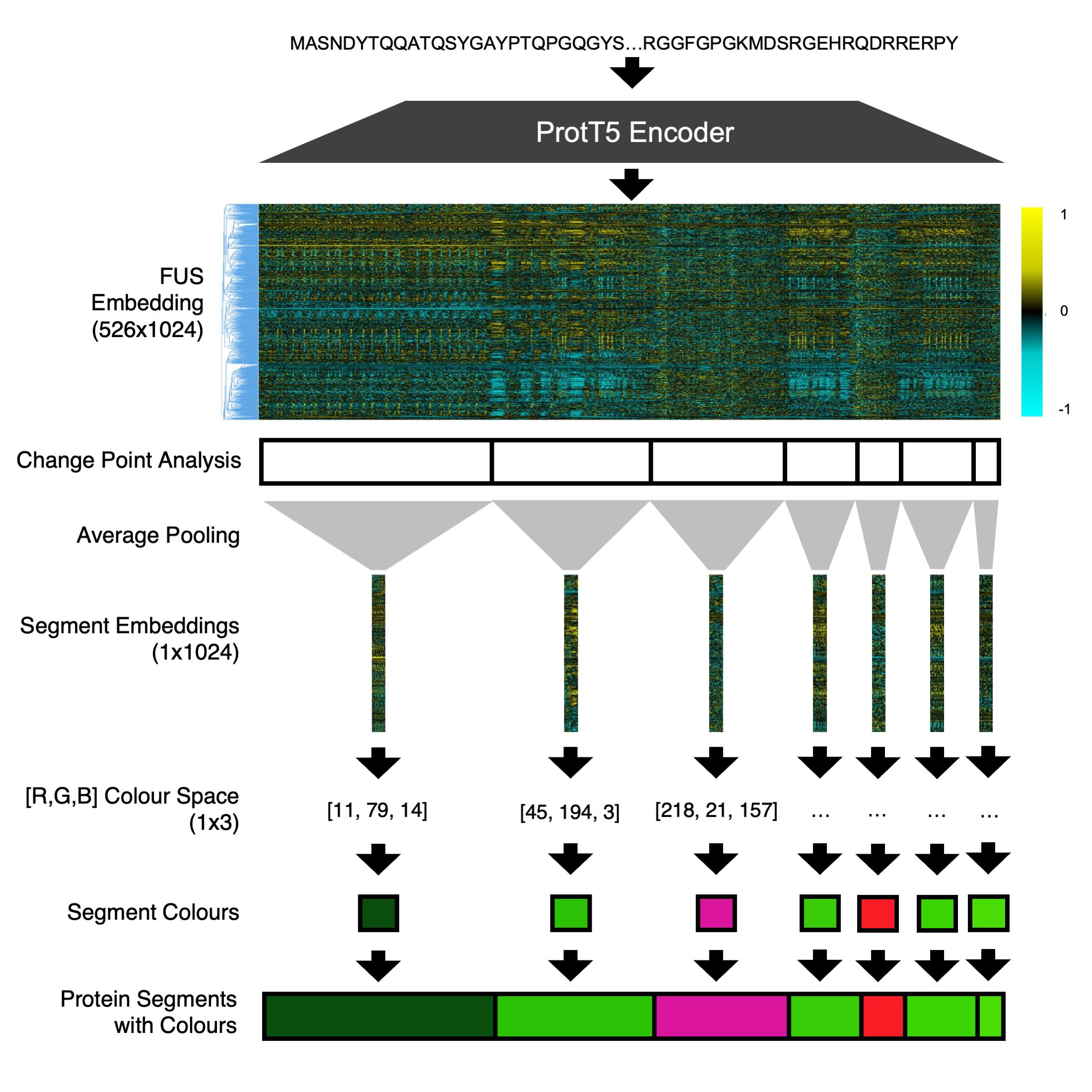

Supplement: S1 Fig — From top to bottom, starting with a protein sequence, we pass it to the ProtT5 encoder which produces a residue-level protein embedding, where the size of the embedding is 1024 by the length of the protein. Next, we perform a change point analysis which identifies boundaries (or break points) along the proteins sequence, which define the segments of the protein. The portion of the residue-level embedding that is contained within these segments is average pooled into “segment embeddings”. Then the segment embeddings are reduced and scaled to represent RGB colours. Last, the segments defined by the change point analysis shown with the ZPS colours to visualize the segment boundaries and corresponding segment embeddings. Note, this figure was designed as a simplified depiction of what happens between panel A and B of Fig 1, it does not show the actual boundaries and ZPS colours of FUS (though they appear similar). (TIFF) [file pcbi.1012929.s006.tiff]

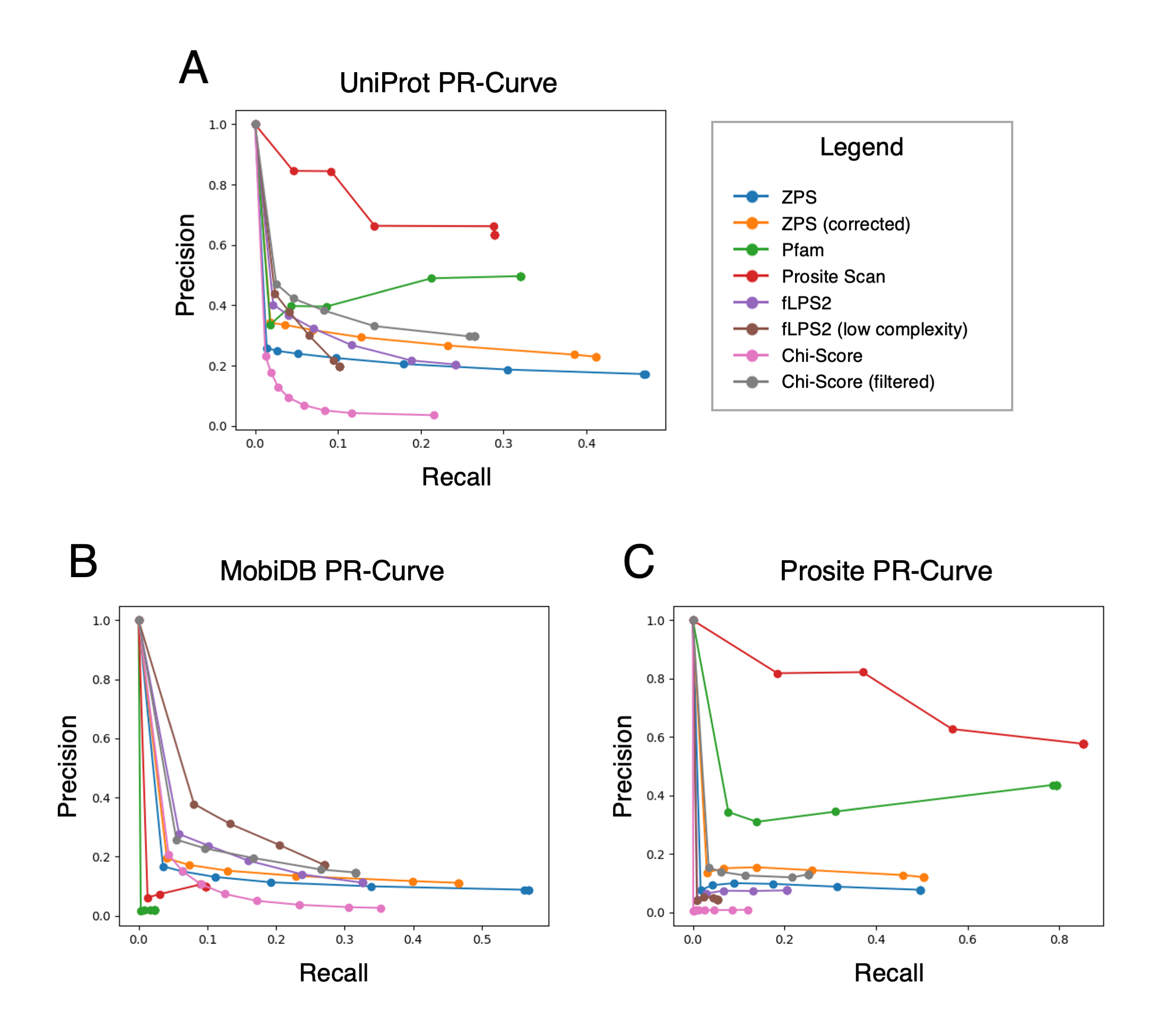

Supplement: S2 Fig — (TIFF) [file pcbi.1012929.s007.tiff]
